# From Patient Liver Tissue to Organoids: Establishment of a Translational Platform Using Healthy, Steatotic, and Cirrhotic Tissue Sources

**DOI:** 10.3390/cells15050432

**Published:** 2026-02-28

**Authors:** Robert F. Pohlberger, Katharina S. Hardt, Mark P. Kühnel, Julian Palzer, Johanna Luisa Reinhardt, Oliver Beetz, Felix Oldhafer, Franziska A. Meister, Katja S. Just, Sarah K. Schröder-Lange, Danny Jonigk, Florian W. R. Vondran, Ralf Weiskirchen, Thomas Stiehl, Anjali A. Roeth

**Affiliations:** 1 Department of General, Visceral, Pediatric, and Transplant Surgery, RWTH Aachen University Hospital, D-52074 Aachen, Germany; rpohlberger@ukaachen.de (R.F.P.); jpalzer@ukaachen.de (J.P.); johanna.reinhardt@rwth-aachen.de (J.L.R.); obeetz@ukaachen.de (O.B.); foldhafer@ukaachen.de (F.O.); fmeister@ukaachen.de (F.A.M.); fvondran@ukaachen.de (F.W.R.V.); 2 Institute of Molecular Pathobiochemistry, Experimental Gene Therapy and Clinical Chemistry (IFMPEGKC), RWTH Aachen University Hospital, D-52074 Aachen, Germany; khardt@ukaachen.de (K.S.H.); saschroeder@ukaachen.de (S.K.S.-L.); rweiskirchen@ukaachen.de (R.W.); 3 Institute for Pathology, RWTH University Hospital Aachen, D-52074 Aachen, Germany; mkuehnel@ukaachen.de (M.P.K.); djonigk@ukaachen.de (D.J.); 4 Institute for Clinical Pharmacology, RWTH Aachen University Hospital, D-52074 Aachen, Germany; kjust@ukaachen.de; 5 Institute for Computational Biomedicine and Disease Modelling, RWTH Aachen University Hospital, D-52074 Aachen, Germany; tstiehl@ukaachen.de; 6 Department of Science and Environment, Roskilde University, DK-4000 Roskilde, Denmark

**Keywords:** liver organoids, patient-derived organoids, steatotic liver disease, cirrhosis, MASLD, 3D cell culture, multiplex immunofluorescence, mathematical growth modeling

## Abstract

**Highlights:**

**What are the main findings?**

- 45 patient-derived liver organoids from healthy, steatotic, and cirrhotic tissue were successfully established with an 82% initiation success rate after optimizing the Broutier protocol.
- The organoids exhibited high proliferation, co-expression of hepatocyte markers (Albumin and HNF4α) and cholangiocyte markers (CK19), sporadic LGR5 positivity, and a biphasic growth pattern, as revealed by mathematical modeling.

**What are the implications of the main findings?**

- These organoid cultures provide a physiologically relevant 3D platform for dissecting the mechanisms of MASLD progression and comparing disease phenotypes across patient cohorts, overcoming the limitations of traditional 2D cultures.
- The combination of molecular profiling and quantitative growth modeling paves the way for precision drug testing, integration of systems biology, and personalized therapeutic strategies in liver disease research.

**Abstract:**

Metabolic dysfunction-associated steatotic liver disease (MASLD) and its consequences represent a growing global health burden that urgently requires physiologically relevant in vitro models beyond conventional 2D culture systems. In this study, we report the successful establishment of 45 patient-derived liver organoid lines. These organoids were generated from healthy, steatotic and cirrhotic tissues collected from 207 liver surgeries at RWTH University Hospital Aachen, with an initiation success rate of 82%. The organoids were propagated for at least six passages using an optimized protocol. Multiplex immunofluorescence analysis revealed highly proliferative structures with approximately 40% Ki-67-positive cells expressing hepatocyte (Albumin and HNF4α) and cholangiocyte (CK19) markers. Intermittent LGR5 staining suggested the presence of liver progenitor cell features. Quantitative PCR results confirmed variable HNF4α expression, indicating inter-patient heterogeneity in differentiation status. Time-lapse imaging combined with mathematical modeling uncovered a biphasic growth dynamic with an initial linear expansion in the first 15 h, followed by exponential growth (doubling time ≈ 20.6 h) between 30 and 72 h. Overall, our workflow produced genetically and phenotypically stable liver organoids that recapitulate essential features of various hepatic conditions. This provides a solid foundation for disease modeling, potential drug testing, and quantitative systems biology.

## 1. Introduction

The liver is an essential digestive organ with several hundred physiological functions, including metabolic functions, immunological functions, and detoxification [1,2]. On a cellular level, the liver is a complex structure composed of hepatocytes, cholangiocytes, stellate cells, Kupffer cells, and liver sinusoidal endothelial cells [2]. Fatty liver and its subsequent liver diseases, such as cirrhosis and hepatocellular carcinoma (HCC), are among the most prevalent and impactful health issues globally. These diseases affect more than 30% of the global population [3,4,5,6]. Metabolically dysfunction-associated steatotic liver disease (MASLD) is a spectrum of conditions, ranging from metabolically associated steatohepatitis (MASH), characterized by steatosis, inflammation, and hepatocyte ballooning, to cirrhosis, and may result in HCC [5,7,8]. It is closely associated with the metabolic syndrome, which includes cardiometabolic criteria such as obesity, insulin resistance, glucose intolerance, and type 2 diabetes mellitus [9,10,11]. MASLD is defined as a steatotic liver disease associated with at least one of the previously described cardiometabolic criteria [12].

Exploring new options for understanding liver diseases is urgently needed, as 2D cell cultures do not adequately mimic cell characteristics such as polarity, morphology, cell–cell interaction, and the (tumor) microenvironment [13,14]. 3D models, such as state-of-the-art patient-derived organoids, are among the most effective methods to overcome these challenges [15,16]. Organoids are clusters of cells with self-renewal and self-organization properties that grow in a 3D extracellular matrix and mimic the characteristics and functionality of the tissues from which they are derived, such as a healthy liver, cholangiocarcinoma, and hepatocellular carcinoma [14,16,17]. This unique ability of organoids to mimic the in vivo environment, as well as their ability to retain genetic and phenotypic characteristics, makes them valuable tools for understanding the complex nature of hepatic diseases [18,19]. These models may then be used for finding new ways to treat hepatic diseases.

In this study, we demonstrate the generation of patient-derived liver organoids obtained from surgeries performed in our hospital, with a focus on pitfalls that must be taken into account when establishing an organoid bank. Additionally, we developed a platform to investigate the organoids on the protein and mRNA level using multiplex immunohistochemical imaging, as well as quantitative PCR (qPCR) [20].

## 2. Materials and Methods

### 2.1. Tissue-Derived Liver Organoids

Human liver tissue was obtained from patients who underwent liver surgery between January 2024 and August 2025 at the Department of General, Visceral, Pediatric and Transplant Surgery of the RWTH University Hospital Aachen. Patients were screened to ensure they met our criteria. Before surgery, all patients were required to sign an informed consent for the use of their tissue for research purposes. The study was approved by the Institutional Review Board (IRB) (vote number EK206/09) beforehand. Surgeries were only included if a specific amount of tumor-free tissue could be obtained without affecting pathological examination. Resections were performed for colorectal liver metastasis (CRLM), hepatocellular carcinoma (HCC), cholangiocellular carcinoma (CCC), or transplant cases.

**Figure 1:**
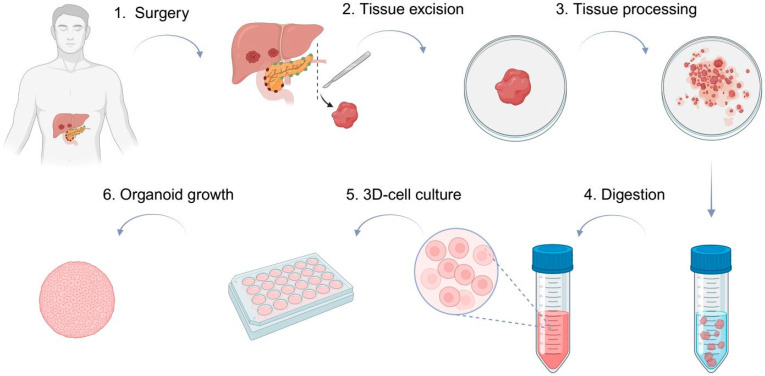
Procedure for producing liver organoids from patient tissue. Created in BioRender. Meister, F. (2026) https://BioRender.com/jbvxejl.

The protocol developed by Broutier et al. was adapted for our specific needs through multiple pilot tests (Figure 1) [21,22].

In summary, the patient’s tissue was minced into small pieces and washed twice with ice-cold Dulbecco’s Modified Eagle Medium (DMEM) containing 4.5 g/L glucose (#11995965, Gibco, Carlsbad, CA, USA) to remove any remaining fat or blood cells. After removing the supernatant, the tissue was washed with digestion media (advanced DMEM/F12 (#12634010, Gibco), 2.5 mg/mL collagenase D (#11088858001, Roche Diagnostics, Rotkreuz, Switzerland), and 0.1 mg/mL DNase I (#10104159001, Roche Diagnostics) and incubated in a shaking incubator (Infors AG, Bottmingen, Switzerland). The cell suspension was then filtered through a 70 μm cell strainer and washed with ice-cold DMEM. The pellet was resuspended in Geltrex (50 μL/24-well) (#A1413202, Gibco) and seeded as domes in 24-well plates for suspension (#662102, Greiner Bio-One GmbH, Kremsmuenster, Austria). Cells were not counted because there was a large number of erythrocytes and debris left in the pellet, which were not capable of forming organoids. The droplets were solidified (37 °C incubator, 30 min) before adding the organoid initiation medium (HepatiCult™ Human Organoid Initiation Medium (#100-0384, Stemcell Technologies, Vancouver, BC, Canada), 10 mM Rho kinase inhibitor (#ab120129, Abcam, Cambridge, UK), 500 µL per well). The media was changed every three days. On day 7, the media was switched to 500 μL per well of the organoid growth medium (HepatiCult™ Human Organoid Growth Medium (#100-0385, Stemcell Technologies). When the culture reached a high organoid density or single organoids exceeded 1 mm in diameter, passaging was performed (1:1 ratio). Here, the cell density in the droplet is approximately 20% at the time of seeding. The Geltrex domes were removed from the wells by scraping, transferred to a tube prefilled with basal medium (advanced DMEM/F12, GlutaMax (#35050061, Gibco), HEPES (#15630056, Gibco), Meropenem (#10170648, Eberth Arzneimittel, Ursensollen, Germany). The organoid suspension was centrifuged for 5 min at 140× *g* and 4 °C (Eppendorf Centrifuge 5810 R, Hamburg, Germany), and the supernatant was reduced to a volume of up to 2 mL. The pellet was then fragmented using a 22-gauge cannula syringe (3–5 times), split, and centrifuged again under the same conditions. The resulting pellet was cultured following the above-mentioned protocol.

### 2.2. Patients’ Characteristics

The following equations were used for calculating the patients’ characteristics:

(1) $$\mathrm{BMI}\;\left(\frac{\mathrm{kg}}{m^{2}}\right)=\;\frac{\mathrm{weight}\;(\mathrm{kg})}{{\left[\mathrm{height}\;(m)\right]}^{2}}$$

(2) $$\mathrm{FIB-4}=(\mathrm{Age}\;(\mathrm{years})\;\times \;\mathrm{AST}\;(U/L))/(\mathrm{Platelet}\;\mathrm{count}\;(10^{9}/L)\;\;\times \;\sqrt{\left(\mathrm{ALT}\;(U/L))\right)}$$

(3) $$\mathrm{MELD}=3.78\;\times \;\ln\left[\mathrm{bilirubin}\;\left(\frac{\mathrm{mg}}{\mathrm{dL}}\right)\right]+11.2\;\times \;\ln\left[\mathrm{INR}\right]+9.57\;\times \;\ln\left[\mathrm{creatinine}\;\left(\frac{\mathrm{mg}}{\mathrm{dL}}\right)\right]+6.43$$

(4) $$\mathrm{MASLD-score}=-1.675+0.037\;\mathrm{age}\;\left(\mathrm{years}\right)+0.094\;\times \;\mathrm{BMI}\;\left(\frac{\mathrm{kg}}{m^{2}}\right)+0.99*\frac{\mathrm{AST}\;\left(\frac{U}{L}\right)}{\mathrm{ALT}\;\left(\frac{U}{L}\right)}-0.013\;\times \;\mathrm{thrombocytes}\;\left(\frac{10^{3}}{\mu L}\right)-0.066\;\times \;\mathrm{Albumin}\;\left(\frac{g}{L}\right)$$

### 2.3. Immunohistochemical Analysis

#### 2.3.1. Sample and Serial Section Preparation for Formalin-Fixed Paraffin Embedded Blocks

Organoids were fixed with formalin (1 h, 500 µL/24-well). After removing the formalin, the domes were washed with Phosphate-Buffered Saline (#10010023, Gibco). Hematoxylin solution was added to visualize the nucleus. The wells were then filled with 2 % agarose (wide-range, #11406.02, Serva, Heidelberg, Germany) solution and left to solidify before being removed from the well plate. The fixed blocks were embedded in paraffin and cut into 2.5 µm serial sections using a microtome.

#### 2.3.2. Hematoxylin-Eosin Staining

The serial sections were stained first with hematoxylin followed by counterstaining with eosin.

#### 2.3.3. Immunofluorescence Multiplex Imaging

For immunofluorescence multiplex imaging, the following antibodies were used after establishing specificity on controls: mouse Albumin (1:400, #ab236492, Abcam), mouse CK19 (1:600, #ab7755, Abcam), mouse HNF4α (1:500, #ab41898, Abcam), mouse Ki67 (1:500, #M7240, Dako, Santa Clara, CA, USA), mouse LGR5 (1:500, #TA503316, Origene, Rockville, MD, USA), rabbit collagen I (1:400, #CL50151AP, Cedarlane, Burlington, Canada), and rabbit α-SMA (1:200, #ab124964, Abcam). After removing paraffin, antibody retrieval was performed using citrate buffer (#S1699, Target Retrieval Solution, Dako, Santa Clara, CA, USA) in a pressure cooker system (Decloaking Chamber, Biocare Medical, Pacheco, CA, USA), followed by blocking non-specific binding sites (#S3022, Dako). The sections were then incubated at room temperature for 30 min with the primary antibodies. Subsequently, an HRP-conjugated secondary antibody (#MP-7452-15, ImmPRESS HRP, Vector Laboratories, Newark, NJ, USA) was applied. Visualization in fluorescence imaging was achieved using the TSA-Plus Cyanine 5 System (#NEL745001KT, Akoya Biosciences, Marlborough, MA, USA), DAPI (#H-1500, Nuclei staining, Vector Laboratories), and the Opal fluorophores Opal 480 (#FP150001KT), Opal 520 (#FP1487001KT), Opal 570 (#FP1488001KT), Opal 650 (#FP1496001KT), Opal 780 (#FP1501001KT) obtained from Akoya Biosciences (Marlborough, MA, USA). If more than one antibody was used, the starting at the blocking step was repeated.

Fluorescence imaging was conducted using Tissue FAXS (TissueGnostics, Vienna, Austria) and quantitative image analysis was performed with StrataQuest Analysis Software (version 7, TissueGnostics). All antibodies used had been previously established.

### 2.4. Quantitative Real Time PCR

Total RNA was isolated using a RNeasy Mini kit (#74104, Qiagen, Hilden, Germany) following the manufacturer’s instructions. RNA purity, quantity, and quality were assessed with a microplate reader (Biotek Synergy HT, Winooski, VT, USA). DNA digestion was carried out using Ambion DNase I and DNase I Buffer (#AM2224, Invitrogen, Waltham, MA, USA). cDNA synthesis was performed with the iScript cDNA Synthesis Kit (#1708840, Bio-Rad, Hercules, CA, USA) according to the manufacturer’s protocol. cDNA was amplified with PowerTrack SYBR Green Master Mix (#A46012, Thermo Fisher, Waltham, MA, USA). As a positive control cDNA prepared from mRNA isolated from a human hepatoblastoma cell line (HepG2, available at ATCC), incubated at 37 °C in a humidified atmosphere containing 5% CO_2_, was used. qPCR was performed using an Applied Biosystems 7500 Real-Time PCR System (Waltham, MA, USA) for *HNF4a* and *MKI67* and an Applied Biosystems 7300 Real-Time PCR System (Waltham, MA, USA) for *LGR5* and *Alb*. Expression levels were normalized using the housekeeping gene *36B4* qPCR primer pair (self-designed) and *GAPDH* (self-designed, NM_001289726.2). The human *HNF4a* qPCR primer pair (#HP200437, NM_000457, Origene), human *MKI67* (#HP206104, NM_002417.5, Origene), human *LGR5* (NM_001277226.2, [23]), and human *ALB* (NM_000477.7, [24]) were used. Sequences are listed in Table 1. C_t_ values (technical triplicates) were normalized to the housekeeping gene. Relative expression levels were calculated using the ΔΔCT method [25], normalized to *36B4* expression in *HNF4A* and *MKI67*, and normalized to *GAPDH* expression in *LGR5* and *ALB*.

### 2.5. Mathematical Modeling

The surface area of the organoid was calculated as 4πr^2^, where r represents the radius of the organoid manually extracted from time-lapse imaging (ImageXpress Pico, Molecular Devices, San José, CA, USA). The surface was used for comparison with monolayer cultures. A linear model y = a × t + b was fitted to the surfaces calculated during the initial 15 h of the experiment, with time denoted as t and surface area denoted as y. The slope a and intercept b are considered free parameters. For the surfaces calculated between hour 30 and hour 72 of the experiment, an exponential growth model, y = a × exp(b × (t − 30 h)) was used. This model includes two free parameters: the surface area at t = 30 h (a) and the proliferation rate (b). A least square cost function is minimized to measure the distance between the data and the model, with all measurements given equal weight. The linear model was fitted using the fitlm function from MATLAB R2024b (Mathworks, Natick, MA, USA), while the exponential model is fitted using fmincon with an interior-point method.

### 2.6. Statistical Analysis

All statistical analyses were conducted using GraphPad Prism 10.6.0. Statistical significance was set at *p* < 0.05, with levels indicated by asterisks (* *p* < 0.05, ** *p* < 0.01, *** *p* < 0.001, and **** *p* < 0.0001).

## 3. Results

### 3.1. Patient’s Characteristics

Patients had a male-to-female ratio of 26:19 (58.42 %), with an average age of 66.6 ± 9 years and a mean BMI of 27.7 ± 4.6 kg/m^2^ (Table 2). Most patients were classified as American Society of Anesthesiologists (ASA) III (N = 28), while fewer were classified as ASA II (N = 9) or ASA IV (N = 7). The mean MASLD score was −0.69 ± 2.38, while the mean Fibrosis-4 (FIB-4) score was 5.45 ± 8.88, and the mean Model for End-Stage Liver Disease (MELD) score was 9.6 ± 7.1, indicating a heterogeneous range of liver function and fibrosis severity [26,27,28].

### 3.2. Patient-Derived Organoids

Between January 2024 and August 2025, we successfully created 45 organoid lines out of 55 attempts (an 82% success rate, Table 3) using various non-cancerous liver tissues from a total of 207 liver surgeries conducted at our clinic.

**Figure 2:**
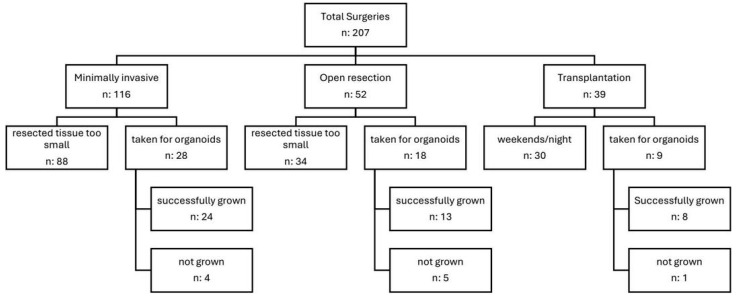
Number of surgeries required for organoid generation. n: numbers.

These surgeries included cases of hepatocellular carcinoma (HCC), cholangiocarcinoma (CCC), colorectal liver metastasis (CRLM), and cirrhosis (Figure 2).

In total, 207 consecutive patients who underwent liver resections were divided into 116 minimally invasive procedures, 52 open resections, and 39 transplantations. Among the 88 cases of minimally invasive surgery and 34 cases of open resection, the resected tissue was too small to generate organoids. Despite these challenges, we successfully created organoids from 28 patients who underwent minimally invasive surgery and 18 patients with open resection, alongside pathological diagnosis. Overall, we achieved an initiation success rate of over 80%, which remained stable across four passages (82%) but declined after six passages (76%). We were able to consistently culture patient-derived liver organoids from healthy (N = 20), steatotic (N = 12), and cirrhotic tissues (N = 13), including samples obtained through minimally invasive procedures, open surgical resections, and transplant surgeries, under controlled laboratory conditions.

Our study revealed that various external factors, such as small resected tissue size or surgeries conducted at night or on weekends, led to unusable tissue. Approximately 20% of cases did not result in organoid growth. In three of these non-growing cases, contamination with intrahepatic bile duct bacteria was observed. To address this issue, we transitioned from standard penicillin/streptomycin antibiotics to the broad-spectrum meropenem antibiotic, which effectively eliminated contamination in most experiments. Despite the switch to meropenem, multidrug-resistant bacteria were encountered in one experiment.

**Figure 3:**
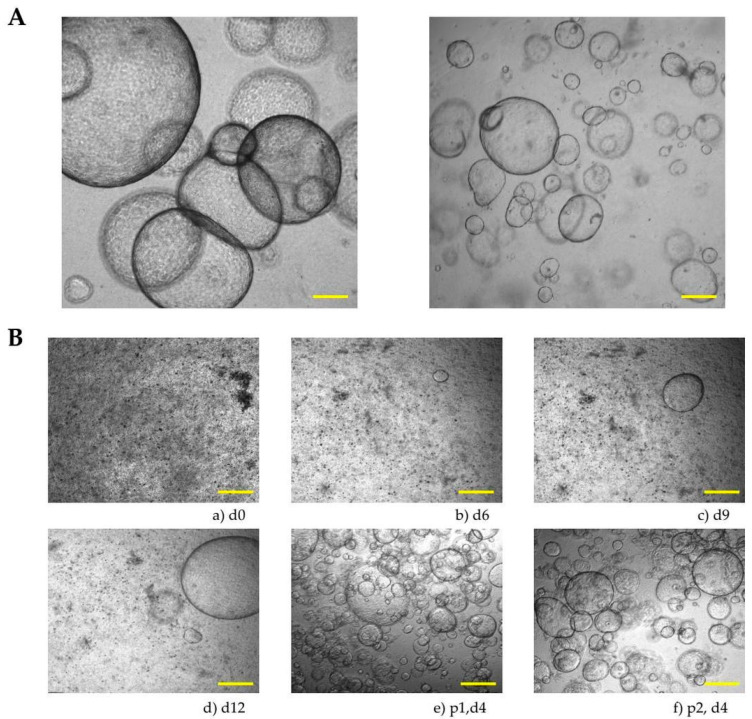
Patient-derived human organoid culture. (**A**) Representative images of a human organoid culture. (**B**) The growth progression of the patient-derived human organoid culture is shown at different time points: day 0, day 6, day 9, day 12, day 4 (after passage 1), day 4 (after passage 2). Scale bars, 200 µm.

All patient-derived liver organoids from our study exhibited the same morphology (Figure 3A). In the early stages, we observed small, dense cell aggregates that grew into larger organoids with well-defined borders. The organoids were cultured under stable conditions for several weeks, allowing for experiments such as immunohistochemistry or qPCR (Figure 3B).

### 3.3. Immunohistochemical Multiplex Analysis

All organoids were fixed in FFPE blocks for analysis of their cellular composition. Using multiplex immunohistochemistry, we applied DAPI and five selected markers to demonstrate the distribution of antibodies in the organoids. Specifically, we used a proliferation marker (Ki-67), two hepatocyte markers (Albumin and HNF4α), a cholangiocyte marker (CK19), a liver stem cell marker (LGR5), and two fibrosis markers (Collagen I and SMA).

**Figure 4:**
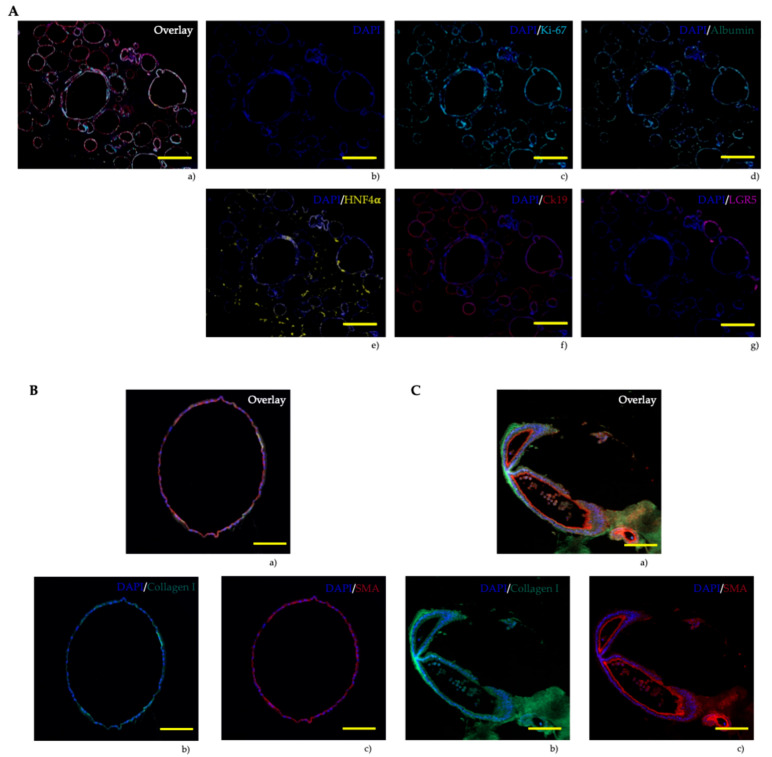
Immunofluorescence imaging of patient-derived organoid culture. (**A**) (**a**) Overlay of all markers, (**b**) DAPI (blue, nuclear staining), (**c**) Ki-67 (cyan, proliferation marker), (**d**) Albumin (green, hepatocyte marker), (**e**) HNF4α (yellow, hepatocyte marker), (**f**) CK 19 (red, cholangiocyte marker), (**g**) LGR5 (pink, liver-specific stem-cell marker). (**B**) Healthy patients’ organoids (**a**) Overlay of all markers, (**b**) Collagen I (green, collagen marker), (**c**) SMA (red, hepatic stellate cells and myofibroblasts). (**C**) Cirrhotic patients organoids (**a**) Overlay of all markers, (**b**) Collagen I (green), (**c**) SMA (red). Scale bars: 200 µm.

**Figure 5:**
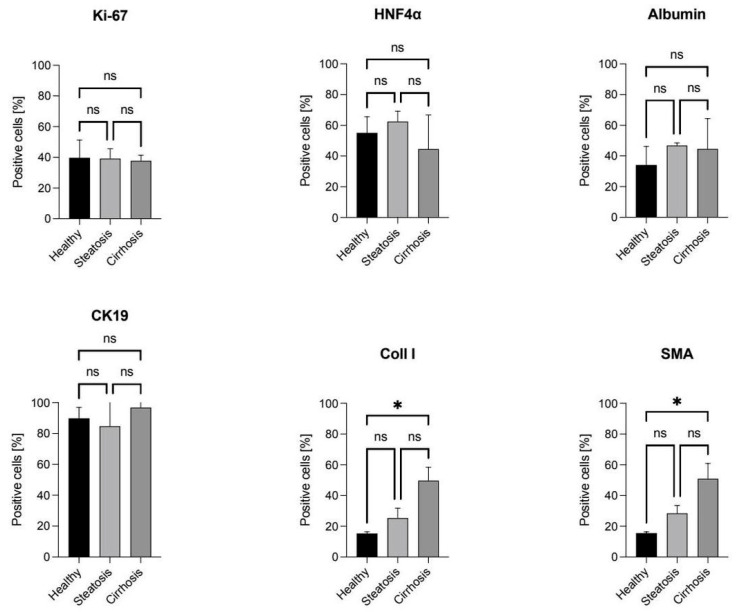
Analysis of antibody distribution in liver organoids derived from healthy, steatotic, and cirrhotic patients. The antibodies used were Ki-67, Albumin, HNF4α, CK19, Collagen I, and SMA. The bars in the figure represent the mean ± SD from three technical replicates (N = 3). An ordinary one-way ANOVA was conducted for statistical analysis (ns: non-significant, * *p* < 0.05).

Patient-derived liver organoids displayed high proliferation rates, with nearly equal numbers of positive cells in healthy, steatotic, and cirrhotic organoids (approximately 40%) (Figure 4), consistent with their rapid growth. Interestingly, Albumin was expressed in Ki-67-positive cells, which is atypical for the liver, where Albumin is typically expressed in differentiated hepatocytes [29,30]. Healthy tissue organoids showed 35% positive cells, while steatotic or cirrhotic tissue organoids showed slightly higher percentages (around 45%) (Figure 5).

**Figure 6:**
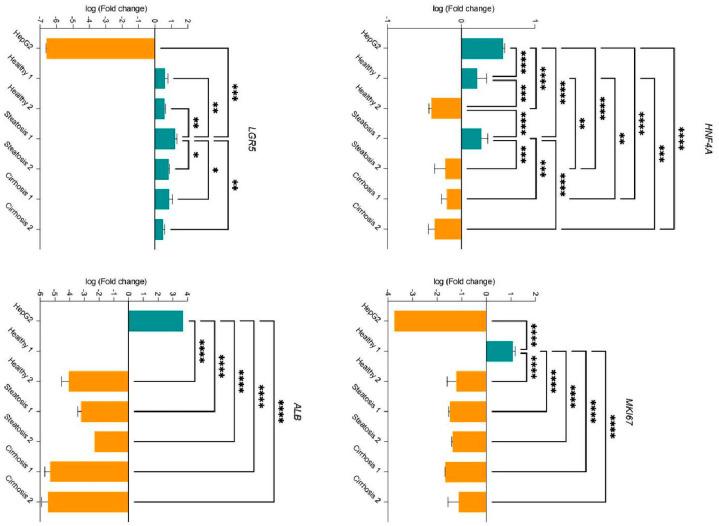
Relative expression of *HNF4A*, *MKI67*, *LGR5*, and *ALB* in human organoids as determined by qPCR. The bars represent the mean ± SD (N = 3). An ordinary one-way ANOVA was conducted for statistical analysis (* *p* < 0.05, ** *p* < 0.01, *** *p* < 0.001, and **** *p* < 0.0001).

Cells that did not test positive for proliferation showed expression of HNF4α, a characteristic hepatocyte marker [31,32]. Healthy and steatotic tissue organoids had around 60% HNF4α-positive cells, while cirrhotic tissue organoids showed lower percentages (approximately 50%). Additionally, the organoids were assessed for the presence of cytokeratin 19, a marker of cholangiocytes [33]. Our findings indicated that the organoids also exhibited cholangiocyte-specific expression patterns, suggesting liver progenitor cell (LPC) characteristics [34]. However, some parts of the organoids showed LGR5, making it difficult to definitively classify them as either stem cells or mature cells (Figure 4A). To support our findings, quantitative PCR was performed for LGR5. Additionally, Collagen I and α-SMA were stained to evaluate if the organoids retain their specific properties (healthy, steatotic, or cirrhotic) from the tissue from which they were derived. Here, we observed that both antibodies increase from organoids derived from healthy tissue to those derived from steatotic or cirrhotic tissue (Figure 5). To validate these results, quantitative real-time PCR was conducted to confirm whether the organoids exhibited comparable RNA expression patterns (Figure 6).

### 3.4. Quantitative Real-Time PCR

To demonstrate the feasibility of RNA detection in organoids using qPCR, we assessed the expression of *HNF4A*, *MKI67*, *LGR5*, and *ALB* in human organoid samples (Figure 6). Organoids derived from both healthy and steatotic tissue displayed significant up- and downregulation of *HNF4A*. However, some samples showed a particularly pronounced downregulation with a fold change of 0.50. Interestingly, organoids from cirrhotic tissue exhibited consistent downregulation of *HNF4A* expression. Additionally, *MKI67* was slightly downregulated in most samples, with only one healthy organoid showing upregulation of proliferation. To further support our findings in Figure 4, we also assessed *LGR5*; the samples showed slightly upregulated levels in all organoid samples. The presence of *LGR5* is consistent with the findings on protein levels. *ALB* was downregulated in all analyzed samples. For one organoid specimen, Albumin transcripts were below the limit of detection in the qPCR assay. 

### 3.5. Computer Simulation

**Figure 7:**
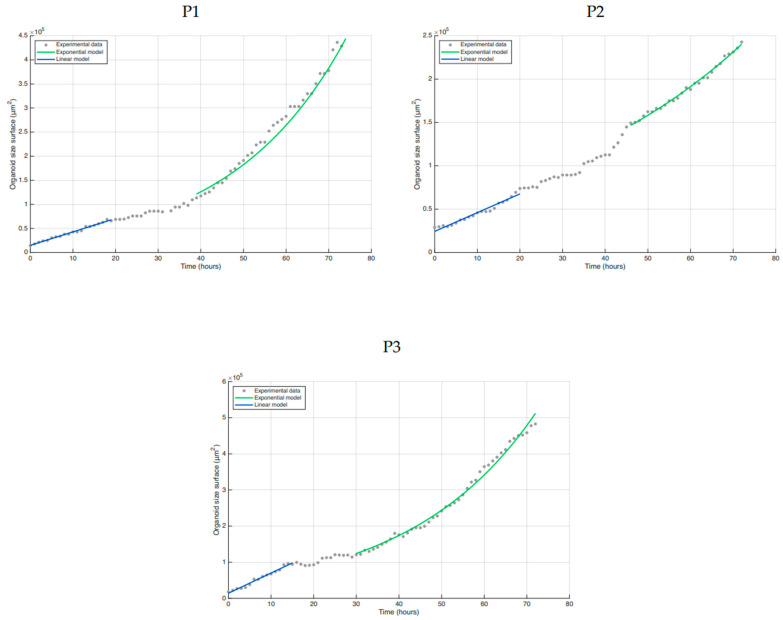
Growth dynamics of organoid surfaces (N = 9 from three individual patients). The initial phase can be accurately described by a linear model (blue). After a transition phase, an exponential model is fitted to the data (green). Organoid surfaces were calculated based on the radius and are indicated in gray.

To better understand how the dynamics of organoid growth change during the experiment, we monitored the radii of individual organoids over time. Assuming a constant and uniform cell size, the surface area of a single-layered organoid is proportional to its cell number. In Figure 7, we observe a multi-phase growth dynamic.

The initial growth phase up to approximately 20 h is linear. Afterwards, a transition phase begins, followed by exponential growth dynamics until the end of the experiment at 72 h. We interpret the transition phase as the period during which cells switch between the two growth modes. The transition phase accounts for less than one population doubling, resulting in approximately a 25% increase in organoid surface area. To further quantify the growth dynamics, we fitted linear and exponential growth models to the respective time intervals. In the initial phase, we observe an increase in surface area by a factor of 3.6 in P1, 1.5 in P2, and 6.9 in P3, corresponding to 3.3 in P1, 2.0 in P2, and 2.8 in P3 population doublings, assuming that the surface change is proportional to the change in cell numbers. If we neglect cell death, the proliferation rate obtained from the exponential model is 0.037 in P1, 0.019 in P2, and 0.034 in P3, corresponding to population doubling times of 25.0 h in P1, 34.6 h in P2, and 20.6 h in P3.

Taken together, the growth dynamics of human liver organoids follow a non-trivial pattern consisting of three multiple phases.

## 4. Discussion

In this study, we demonstrated the production and feasibility of generating liver organoids from patients’ tissue obtained during surgery at our clinic. We achieved organoid initiation rates above 80%. By adapting the Huch Group protocol, liver organoid production from tissues under various conditions (healthy, steatotic, and cirrhotic) can be scaled up to compare patients with different diseases and conditions [21,22]. In this context, the benefits of minimally invasive surgery for patients become clear: less tissue needs to be removed, yet less tissue remains for organoid generation [35]. Brightfield microscopy confirmed the successful formation of three-dimensional organoids from the patient’s liver. Nonetheless, the generation of liver organoids from these three patient groups has limitations. For example, no additional fatty acids were added to the medium in the steatotic group. Future studies must determine the effects this addition might have on healthy liver organoids or the absence of it on steatotic organoids. Furthermore, by incorporating Hoechst staining to differentiate between cells and cell debris, these limitations could be addressed, resulting in a more accurate cell count being included in the protocol.

Additionally, we successfully analyzed organoids at the protein and RNA levels. At the protein level, organoids exhibit high Ki-67 expression, indicating their rapid growth. Furthermore, Ki-67 and Albumin were expressed by the same cells, which is not typical for the liver [36]. In addition to these cells, HNF4α was expressed, demonstrating the hepatic nature of the organoids. Moreover, all organoids show high CK19 expression, supporting the idea that liver organoids display both hepatic and biliary features. LGR5 was not fully expressed in the organoids, confirming previous observations from our laboratory [37]. Quantitative PCR supported this finding. Furthermore, using collagen I and α-SMA, we demonstrated that organoids maintain the disease status of the tissue from which they were derived. However, the varied staining patterns suggest different levels of differentiation within individual organoids, which may reflect distinct cell subpopulations or different stages of maturation. Here, the limitation of having only N = 3 per group becomes evident as more organoid lines need to be analyzed.

Additionally, gene expression analysis using quantitative PCR (qPCR) supported these results. *HNF4a* exhibited variable expression across samples, with some organoids showing upregulation, while others displayed lower expression compared to the reference sample. Overall, the findings suggest a diverse range of *HNF4a* expression levels among the organoid panel, indicating differing differentiation states. Organoids with elevated *HNF4a* expression likely demonstrate a more hepatocyte-like phenotype, whereas those with lower expression may suggest dedifferentiation or an immature state. Furthermore, *LGR5* and *ALB* were investigated in order to support these findings. This variability may reflect differences in maturation or heterogeneity within the cultures, requiring further experiments for a comprehensive understanding of the underlying mechanisms. In conjunction with the immunohistochemistry multiplex results, these data highlight significant heterogeneity among organoids derived from different patients. However, the complete cell composition of the organoids remains unclear, as other liver cell types, such as hepatic stellate cells, sinusoidal endothelial cells, and Kupffer cells, have not yet been examined.

Mechanistic, mathematical, and computational modeling is the first step towards achieving a quantitative understanding of organoid growth kinetics and the related cellular processes. By applying simple growth models to the time dynamics of organoid surface area, we discovered that organoid surface area grows linearly within a specific time frame, with variations observed among patients. If the relative change in organoid surface reflects the relative change in cell numbers, an increase would suggest a high division frequency at the beginning of the experiment.

An alternative explanation for this observation could be that spatial rearrangements of cells or culture-related changes in cell diameter or shape due to culture conditions, in the early phase of the experiment, may contribute to the surface growth. In the later phases, the surface area follows an exponential growth curve, with population doubling times of approximately 20–35 h, as expected under optimal growth conditions in the absence of resource limitations. Further experiments are necessary to comprehend the mechanisms underlying the different growth phases and their transitions.

Overall, our data demonstrate that human liver organoids can be reliably generated from patients’ tissue and maintained in vitro, exhibiting growth patterns, hepatic marker expression, and gene expression profiles that are partly consistent with hepatocyte identity. Further research is needed to fully understand the initiation, growth, and cell composition of tissue-derived liver organoids.

## 5. Conclusions

We have demonstrated that patient-derived liver organoids can be reliably generated from surgical specimens representing healthy, steatotic, and cirrhotic tissues. These organoids can be maintained long-term and analyzed at molecular, cellular, and computational levels. Their high initiation efficiency, which preserves disease-specific phenotypes and reproducible growth kinetics, establishes these organoids as an indispensable bridge between clinical samples and mechanistic research. This paves the way for precision diagnostics and personalized therapeutics in liver disease.

## Figures and Tables

**Table 1:** Primers used in this study.

Gene	Acession No.	Forward primer (5′-3′)	Location	Reverse primer (5′-3′)	Location	Amplicon Size
HNF4A	NM_000457	GGTGTCCATAC GCATCCTTGAC	829–850	AGCCGCTTGAT CTTCCCTGGAT	972–951	144
MKI67	NM_002417.5	GAAAGAGTGGC AACCTGCCTTC	2270–2291	GCACCAAGTTT TACTCATCTGCC	2397–2420	150
LGR5	NM_001277226.2	CGGGAAACGCT CTGACATACAT	576–597	TGAAACAGCTT GGGGGCACATA	758–737	183
ALB	NM_000477.7	GCCAGTAAGT GACAGAGTCAC	1514–1534	TTATAAGCCTA AGGCAGCTTGAC	1871–1849	358
36B4	NM_001002.4	CCTCGTGGAA GTGACATCGT	19–38	ATCTGCTTGG AGCCCACATT	196–177	178
GAPDH	NM_001289746.2	AGCCACATCG CTCAGACAC	57–75	CACCACCCT GTTGCTGTAG	104–122	66

**Table 2:** Characteristics of the patients. All values are shown as the mean ± standard deviation.

Characteristics	Total	Healthy	Steatotic	Cirrhotic
Gender	Male: 26	Male: 11	Male: 9	Male: 7
	Female: 19	Female: 9	Female: 3	Female: 6
Age (years)	66.7 ± 9	68.8 ± 8	66.5 ± 10	64.3 ± 9.8
Heights (m)	1.73 ± 0.09	1.73 ± 0.1	1.74 ± 0.1	1.72 ± 0.07
Weight (kg)	82.6 ± 16.4	80.0 ± 15.9	81.0 ± 17.4	84.9 ± 17.8
BMI (kg/m^2^)	27.5 ± 4.5	26.7 ± 4.2	26.7 ± 4.1	28.6 ± 5.8
ASA score				
II	11	6	5	0
III	28	14	7	7
IV	6	0	0	6
MASLD score	−0.69 ± 2.38	−1.37 ± 2.54	−1.34 ± 1.14	1.34 ± 2.80
FIB-4 score	5.45 ± 8.88	2.43 ± 1.84	2.16 ± 1.64	11.65 ± 12.24
MELD score	9.6 ± 7.1	7.0 ± 3.1	7.67 ± 2.39	16.09 ± 11.27

**Table 3:** Success rate of organoid cultures.

	Used Tissue	Successful Growth (%)	Healthy Tissue (n)	Steatotic Tissue (n)	Cirrhotic Tissue (n)
Enrolment	55	100	22	16	17
Initial organoid formation	45	82	20	12	13
Organoids cultures established (≥passage #2)	45	82	/	/	/
Organoids cultures established (≥passage #4)	45	82	/	/	/
Organoids cultures established (≥passage #6)	42	76	/	/	/

## Data Availability

The datasets generated and/or analyzed during the current study are available from the corresponding author upon reasonable request.

## Abbreviations

The following abbreviations are used in this manuscript:

ASA	American Society of Anesthesiologists
BMI	Body Mass Index
CCC	Cholangiocarcinoma
CK19	Cytokeratin 19
CRLM	Colorectal liver metastasis
DAPI	4’,6-diamidine-2-phenylindole
FFPE	Formalin-fixed paraffin-embedded
FIB-4	Fibrosis-4
HCC	Hepatocellular carcinoma
H&E	Hematoxylin & eosin
HNF4α	Hepatocyte nuclear factor 4 alpha
LGR5	Leucine-rich repeat-containing G-protein-coupled receptor 5
MASH	Metabolic dysfunction-associated steatohepatitis
MASLD	Metabolic dysfunction-associated steatotic liver disease
MELD	Model of end-stage liver disease
qPCR	Quantitative polymerase chain reaction
